# The Effectiveness of Mindfulness-Based Interventions in Enhancing Psychological Well-Being in Athletes: A Scoping Review of Randomised Controlled Trials

**DOI:** 10.3390/healthcare14182899

**Published:** 2026-09-08

**Authors:** Nafih Cherappurath, Halil İbrahim Ceylan, E. Anakha, Saranya T. Satheesan, Deniz Öztürk, Muhammed Ali Thoompenthodi, Masilamani Elayaraja, Rinsa Raj, Raul Ioan Muntean, P. C Firdousiya

**Affiliations:** 1 Department of Physical Education, Amal College of Advanced Studies (Autonomous), Nilambur 679329, Kerala, India; 2 Faculty of Sports Sciences, Atatürk University, Erzurum 25100, Türkiye; 3 School of Philosophy, Psychology and Scientific Heritage, Chinmaya Vishwa Vidyapeeth, Kochi 682016, Kerala, India; 4 Department of Psychology, School of Social Sciences, Vellore Institute of Technology, Chennai 600127, Tamil Nadu, India; 5 Health Services Vocational School, Atatürk University, Erzurum 25240, Türkiye; 6 Department of Physical Education, Malabar College of Advanced Studies, Vengara 676519, Kerala, India; 7 Department of Physical Education and Sports, Pondicherry University, Puducherry 605014, Puducherry, India; 8 Bachelor of Physical Education Centre, Chakkittapara 673526, Kerala, India; 9 Department of Physical Education and Sport, Faculty of Law and Social Sciences, University “1 Decembrie 1918” of Alba Iulia, 510009 Alba Iulia, Romania; 10 Department of Psychology, Amal College of Advanced Studies (Autonomous), Nilambur 679329, Kerala, India

**Keywords:** mindfulness, athletes, psychological well-being, randomised controlled trial, scoping review

## Abstract

**Background/Objectives:** Mindfulness-based interventions (MBIs) have gained increasing attention in sport psychology due to their potential to enhance psychological well-being and performance-related outcomes among athletes. Despite various systematic reviews and meta-analyses examining mindfulness in athletic contexts, broader scoping evidence mapping randomised controlled trials (RCTs) of MBIs targeting psychological well-being among athletes remains limited. This scoping review aims to map and synthesise empirical evidence from RCTs examining MBIs and psychological outcomes among athletes, identify methodological and conceptual gaps, and guide future systematic reviews and meta-analytic investigations. **Methods:** The review followed the Joanna Briggs Institute (JBI) methodology for scoping reviews and was reported according to the PRISMA-ScR guidelines. Searches were conducted across Scopus, Web of Science, and PubMed, initially in March 2026 and updated in April 2026. Data were extracted on study characteristics, participant demographics, intervention features, comparator conditions, outcome measures, and key findings. **Results:** A total of 17 studies met the inclusion criteria and were included in the review. The included studies generally reported beneficial effects of MBIs across psychological well-being, emotion regulation, cognitive functioning, and sport-related psychological outcomes among athletes. Beneficial effects were reported for several outcomes, including mindfulness, mental toughness, emotional intelligence, mental well-being, flow state, self-compassion, psychological flexibility, emotional regulation, attention, life satisfaction, and grit. Reductions in psychological distress, particularly anxiety and stress, were also reported in several studies, although findings for anxiety were not consistent across trials. Findings were mixed for some other outcomes, including self-esteem, acceptance, and depression. Improvements were additionally reported in social relationships, pleasure regulation, and overall psychological functioning. **Conclusions:** The findings suggest that MBIs may offer beneficial effects across multiple dimensions of psychological functioning among athletes, although effects were not uniform across outcomes or interventions. These findings indicate the potential utility of structured MBI programmes within athlete support settings, particularly when delivered by or in collaboration with sport psychologists and mental performance consultants, while highlighting the need for further well-designed RCTs to clarify intervention-specific effects and longer-term outcomes.

## 1. Introduction

Mindfulness, defined as nonjudgmental present-moment awareness, has gained significant traction in sports psychology as a means to enhance athletic performance and well-being. Mindfulness-based approaches in sport are diverse and encompass several distinct yet complementary practices. Focused Attention Meditation (FAM), which involves directing attention toward a selected object such as the breath, emphasises sustained and selective attention, whereas Open Monitoring Meditation (OMM) promotes non-reactive awareness of ongoing experiences and broader attentional monitoring [1,2,3]. These meditation approaches have been theoretically proposed to address different attentional demands in sport; however, their suitability should not be assumed solely based on whether a sport is classified as open- or closed-skill. Additional practices, including body scan techniques and mindful movement, enhance interoceptive awareness and embodied attention. Acceptance and Commitment Therapy (ACT), a key third-wave behavioural approach, further emphasises psychological flexibility, acceptance of internal experiences, and commitment to value-driven actions [3]. Moreover, integrated protocols such as Mindfulness-Acceptance-Commitment (MAC) and Mindful Sport Performance Enhancement (MSPE) combine mindfulness principles with sport-specific psychological strategies to enhance performance and improve mental health outcomes [4,5]. Across these approaches, common core components include present-moment awareness, cognitive defusion, acceptance, self-compassion, and facilitation of flow states [1,2,6,7].

Psychological well-being is a multidimensional construct that extends beyond the absence of mental illness and encompasses positive emotional, psychological, and social functioning [8,9,10,11]. According to the dual-continua model, mental health and mental illness are related but distinct dimensions; therefore, individuals may experience high levels of psychological well-being despite the presence of some psychological distress, highlighting that well-being is not merely the absence of mental illness [10,12,13]. In the sporting context, psychological well-being reflects an athlete’s capacity to experience positive emotions, maintain resilience, regulate emotions effectively, develop a sense of purpose and competence, foster meaningful interpersonal relationships, and cope successfully with the psychological demands of training and competition [14,15,16,17].

The present review is theoretically informed by complementary frameworks of psychological well-being and emotion regulation. First, the hedonic–eudaimonic perspective recognises psychological well-being as encompassing both hedonic dimensions, including positive affect, life satisfaction, and the reduction in psychological distress, and eudaimonic dimensions, including personal meaning, autonomy, personal growth, and positive relationships [18,19,20,21,22]. This distinction is particularly relevant in athletes, as psychological health has often been evaluated primarily through the reduction in symptoms such as anxiety, depression, and burnout [23,24]. Accordingly, the present review considers both the reduction in psychological distress and the development of positive psychological functioning [25]. Second, Gross’s Process Model of Emotion Regulation provides a complementary framework for understanding how mindfulness may influence psychological well-being through emotion-regulatory processes [26,27]. Within athletic contexts, mindfulness-based approaches may facilitate non-judgmental present-moment awareness and acceptance of internal experiences, potentially supporting adaptive emotion regulation. The Acceptance-Based Performance Model further contextualises these processes within sport by emphasising acceptance, mindfulness, and psychological flexibility as mechanisms relevant to athlete functioning and performance [28,29,30]. Together, these frameworks provide a conceptual basis for interpreting the diverse psychological well-being outcomes examined across the included RCTs.

Mindfulness, psychological well-being, and athletic performance are closely related but conceptually distinct constructs in sport psychology. In the present review, mindfulness is treated as a trainable capacity involving present-moment awareness, openness, and a non-judgmental attitude [31,32]. It represents the primary psychological process targeted by mindfulness-based interventions (MBIs) and is considered a process-related construct in this review. Psychological well-being represents the primary outcome domain and is conceptualised across both hedonic and eudaimonic dimensions, consistent with broader models of well-being and the dual-continua perspective [18,22]. Athletic performance is treated as a conceptually distinct, sport-specific outcome that was frequently co-assessed in the included trials but was not the primary target construct of this review. These constructs may be interconnected rather than strictly causal. MBIs may cultivate mindfulness and related processes, such as emotional regulation and attentional control, which may contribute to psychological well-being and, potentially, sport-related outcomes.

The application of MBIs varies according to sport type, competitive level, and the specific psychological and attentional demands of the sporting context. Sport skills are commonly differentiated according to the stability and predictability of the performance environment, with sports such as tennis generally characterised as open-skill activities requiring responses to changing external stimuli, whereas swimming is generally considered a predominantly closed-skill, self-paced activity [33,34,35]. Although FAM and OMM have been theoretically proposed to support different attentional processes, evidence directly demonstrating that one meditation approach is superior for a particular sport-skill category remains limited. Accordingly, the selection and adaptation of mindfulness practices should consider the specific attentional, perceptual, and contextual demands of the sport rather than being determined solely by open- versus closed-skill classification. MBIs have been implemented across a broad spectrum of athletes, ranging from amateur and collegiate participants to elite and national-level performers, often with modifications tailored to developmental stages and sport-specific demands [33,34,35].

The characteristics and delivery of MBIs vary across several dimensions, including total intervention duration, session frequency, session length, and home practice [36,37]. Brief mindfulness sessions have been investigated alongside multi-week intervention programmes, with reported effects varying across psychological, attentional, and performance-related outcomes [37,38,39,40]. However, session duration and total programme duration represent distinct dimensions of intervention exposure and should not be interpreted as equivalent indicators of dose. Although some studies have reported immediate effects following brief mindfulness sessions and beneficial outcomes following longer programmes, the available evidence does not establish that longer interventions necessarily produce larger or more sustained benefits [40,41,42,43]. This interpretation is particularly important because intervention characteristics often vary simultaneously across studies, making it difficult to isolate the contribution of any single dose component [44,45,46]. Delivery formats have included in-person, online, coach-led, and team-based approaches, reflecting the adaptability of MBIs to different athletic contexts and logistical requirements [42,47,48,49]. Accordingly, the present review maps intervention duration, session frequency, session length, and delivery characteristics separately to identify patterns in the available evidence rather than assuming a dose–response relationship.

MBIs demonstrate consistent effectiveness in improving key psychological outcomes relevant to athletic performance. Reductions in competitive anxiety and stress are among the most robust findings, with effects often maintained over time [1,50,51]. Additionally, mindfulness training has been linked to enhanced flow states, which mediate performance optimisation [45,51]. Beyond performance-related variables, MBIs contribute to enhanced self-compassion and improved overall well-being across competitive levels [52,53]. In addition to psychological benefits, MBIs have been associated with favourable physiological adaptations. Reductions in stress-related biomarkers, such as cortisol and alpha-amylase, indicate improved regulation of autonomic and endocrine responses to stress [54,55]. Improvements in sleep quality further support recovery processes and overall athlete health [56,57]. While some evidence suggests reduced injury risk and improved psychological adaptation during injury recovery, findings regarding physiological recovery remain inconsistent [58,59]. Similarly, research on heart rate variability (HRV) has yielded mixed results, highlighting the need for further investigation into the mechanisms of autonomic regulation [60].

Increased understanding of mindfulness mechanisms across different outcomes has led to the development of multiple mindfulness training methods. Existing systematic reviews and meta-analyses have primarily focused on mindfulness training approaches in athletic performance enhancement [1,36,38,44,61,62], athlete burnout [63], psychological adaptation after sports injury [59], competitive anxiety [11], and broader psychological outcomes [45,64,65] among athletes. Although these reviews have contributed substantially to understanding mindfulness in sport, they have generally focused on specific psychological constructs, performance outcomes, individual intervention approaches, or pooled estimates of intervention effects. Furthermore, a recent scoping review by Mascarenhas et al. [4] mapped the implementation of MSPE and its effects on flow, performance, and mental health among athletes, but was restricted to MSPE-related interventions. Consequently, the existing evidence base provides limited mapping of the broader range of MBI approaches, intervention characteristics, comparators, and psychological well-being outcomes examined across RCTs involving athletes.

To date, limited scoping evidence has comprehensively mapped randomised controlled trials (RCTs) examining MBIs and psychological well-being outcomes among athletes. This represents a significant research gap, particularly given the growing number of systematic reviews and meta-analyses in mindfulness and sport psychology. The present scoping review addresses this gap by focusing specifically on RCTs involving athletes and systematically mapping the breadth and characteristics of the available evidence rather than estimating a single pooled effect. It characterises the psychological well-being outcomes investigated, the types of MBIs and their intervention characteristics, including duration, frequency, session length, and delivery format, as well as comparator conditions and athlete populations. This scoping approach is appropriate because the available RCTs encompass heterogeneous intervention approaches, delivery formats, comparators, outcome measures, and athlete populations, making it important to first establish the structure and breadth of the evidence base. The decision-relevant contribution of this review is therefore to identify which interventions, outcomes, populations, and intervention characteristics have been studied; describe patterns in reported findings; and identify evidence gaps that can inform more narrowly defined systematic reviews, meta-analyses, and future primary RCTs. Accordingly, the present review does not seek to establish overall comparative effectiveness or superiority of one MBI approach over another, but to provide an evidence map that can guide subsequent effectiveness-focused synthesis and research.

The identified gaps in the existing literature informed the development of the research questions for this scoping review. Previous evidence syntheses have largely focused on specific psychological outcomes or individual mindfulness programmes rather than providing a comprehensive overview of RCTs evaluating MBIs in athletes. Therefore, the research questions were designed to examine how MBIs are conceptualised and implemented in athletic populations, characterise psychological well-being outcomes and patterns of findings across comparator conditions, describe intervention characteristics in relation to reported outcomes, and map the implementation of sport-adapted and standardised MBI approaches. Collectively, these questions provide a structured framework for mapping the available evidence and highlighting key areas for future research.

Based on these identified gaps, six research questions were formulated for the present scoping review:

1. How are MBIs conceptually defined and described in RCTs involving athletes?
2. How are MBIs operationalised in RCTs, including their intervention type, duration, frequency, session length, delivery format, and instructor characteristics?
3. What psychological well-being outcomes have been examined, and what patterns of findings have been reported regarding the effects of MBIs among athletes?
4. What patterns of psychological outcomes have been reported when MBIs are compared with active control conditions in RCTs involving athletes?
5. What intervention characteristics, including duration, frequency, and session length, have been reported in RCTs examining MBIs among athletes, and what patterns in psychological outcomes are observed across these characteristics?
6. How have sport-adapted and standardised MBI approaches been implemented in RCTs involving athletes, and what psychological outcomes have been reported?

## 2. Methods

This scoping review protocol follows the methodological framework developed by the Joanna Briggs Institute for scoping reviews [66,67], with reporting guided by the PRISMA-ScR [68] checklist (Supplementary S1). The review protocol was developed and preregistered on the Open Science Framework (OSF) prior to the commencement of the review (https://doi.org/10.17605/OSF.IO/JBFE2; accessed on 12 April 2026). The review aims to map and synthesise the available evidence on the effects of MBIs on psychological well-being among athletes.

### 2.1. Eligibility Criteria

Eligibility criteria were based on the PCC framework (Table 1) and were in line with Joanna Briggs Institute guidance. To be included, studies had to involve athletes aged 16 years or older, including trained, competitive, collegiate/varsity, or elite athletes in any sport. The intervention needed to be a mindfulness-based approach, such as Mindfulness-Based Stress Reduction (MBSR), Mindfulness-Based Cognitive Therapy (MBCT), body scan, breath awareness, loving-kindness, or similar practices, delivered as a standalone or sport-adapted programme. Mindfulness had to constitute the central therapeutic or training component, or be an explicitly described and separately identifiable component of a multicomponent intervention. Studies were excluded when mindfulness was mentioned only as a minor, incidental, or nonspecific element without a distinct mindfulness-based practice. For this review, psychological well-being was defined as positive psychological functioning and mental health-related outcomes, including both reduced psychological distress and enhanced adaptive psychological resources. At least one psychological well-being outcome was required for inclusion. Studies involving athletes were eligible regardless of the setting in which the intervention was delivered, with no restriction on geography or country. Only RCTs were included. Only studies published in English were included because of resource and translation constraints. Full criteria are presented in Table 2.

### 2.2. Information Sources and Search Strategy

Searches were initially conducted across Scopus, Web of Science, and PubMed in March 2026 and subsequently updated in April 2026 using the same databases and search strategy to identify eligible records published or indexed since the initial search. Records identified through the updated search underwent the same two-stage screening process, comprising title/abstract screening followed by full-text assessment. A comprehensive search strategy was developed around three primary concepts: (1) mindfulness-based interventions, (2) athlete or sport populations, and (3) RCT designs. Within each concept, related terms were combined using “OR”, while the three concept blocks were combined using “AND”. Parentheses were used to clearly define each conceptual block and ensure that the intended intersection of mindfulness, athlete/sport, and RCT terms was retrieved. The strategy included terms such as mindfulness, MBSR, MBCT, meditation, body scan, breath awareness, present-moment awareness, acceptance-based interventions, athlete, sport, player, RCT, and related terms. The complete database-specific search strategies for Scopus, Web of Science, and PubMed, including the exact search strings and syntax, are provided in Supplementary S2. The strategy was adapted for each database based on its indexing conventions. Reference lists of the included studies were also hand-searched; however, this process did not identify any additional eligible studies. Grey literature was not searched for, as the review was limited to peer-reviewed RCTs to maintain methodological rigour and ensure reproducibility.

### 2.3. Study Selection

**Figure 1:**
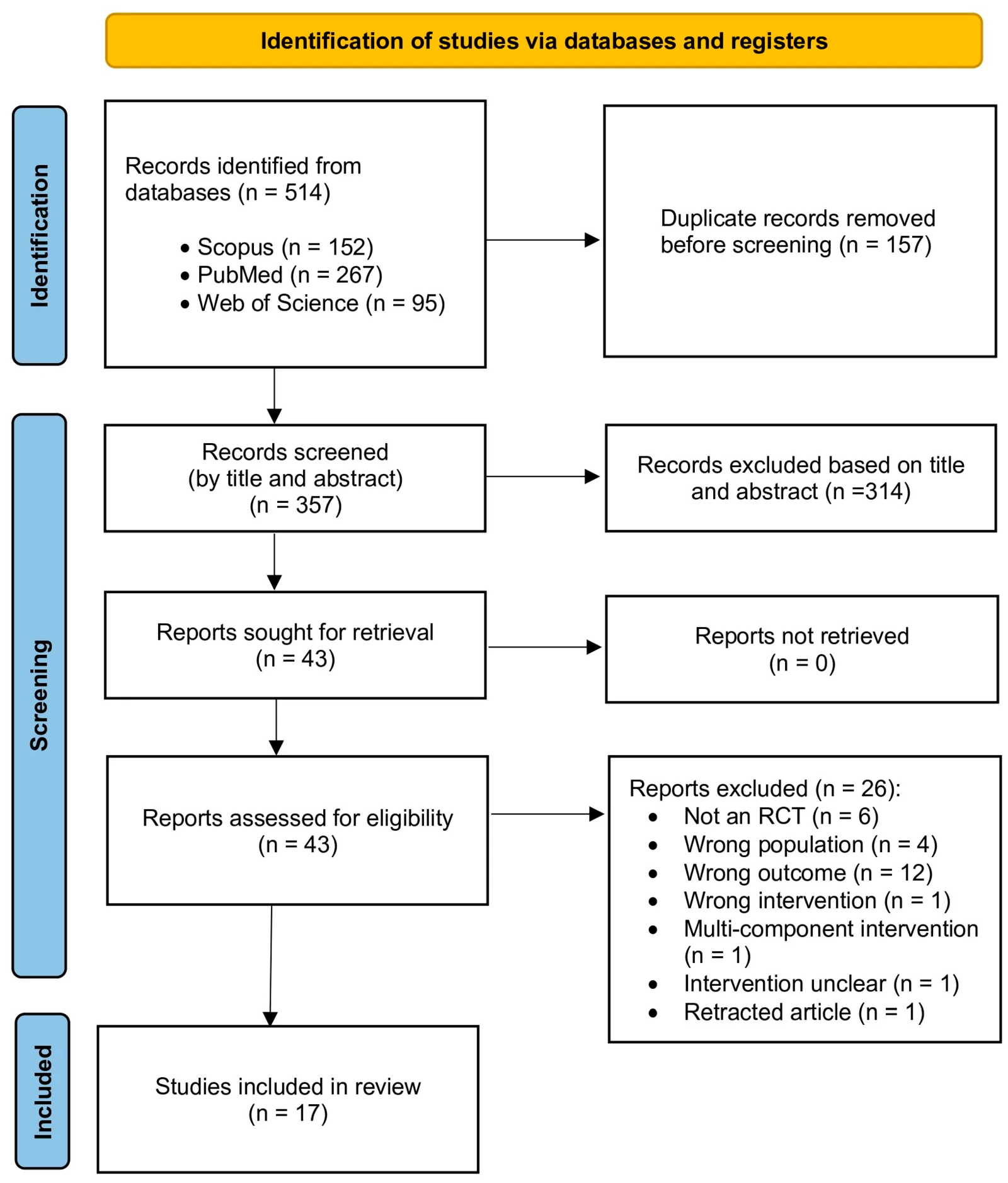
PRISMA flow diagram showing the study selection process.

All records were imported into Microsoft Excel (Microsoft Corporation, Redmond, WA, USA), where duplicate records were identified in Excel using bibliographic fields such as author, title, year, and source, manually checked for consistency, and removed before screening. Two reviewers (N.C. and E.A.) independently screened titles and abstracts and subsequently assessed potentially eligible full texts against the predefined eligibility criteria. Inter-rater reliability was assessed using Cohen’s kappa (κ = 0.87), indicating high agreement between the reviewers. Disagreements between the two reviewers were first resolved through discussion. When the reviewers could not reach an agreement, a third reviewer (S.T.S.) independently reviewed the study and made the final decision. Excel was used to document screening decisions and manage the study-selection process. The whole process is mapped in the PRISMA flow diagram (Figure 1).

### 2.4. Extraction and Charting the Data

Two reviewers (N.C. and E.A.) independently extracted data from the included studies using a data-extraction form developed in Microsoft Excel (Microsoft Corporation, Redmond, WA, USA). Following independent extraction, the reviewers compared the extracted data and resolved discrepancies through discussion. When discussion could not be reached, a third reviewer (S.T.S.) was consulted to adjudicate the discrepancy. The extracted data contained study characteristics (author, year, country), participant details (age, sex, sport type, sample size, competitive level), study design, intervention type, duration, frequency, session length, delivery mode, outcome measures, and key findings (Table 3).

### 2.5. Data Synthesis

The extracted data were synthesised descriptively and narratively. Outcomes were grouped into broad psychological domains, and findings were summarised by intervention characteristics, comparator conditions, and assessment time points, with follow-up findings. Active and passive controls were classified according to the nature of the comparator. Positive effects were defined primarily by statistically significant between-group improvements, with within-group changes considered when between-group comparisons were unavailable. Non-significant and mixed findings were also reported. The six research questions were addressed through descriptive mapping. No quantitative effects were pooled, and no dose–response or comparative-effectiveness conclusions were inferred from cross-study comparisons.

## 3. Results

### 3.1. Search Results

A total of 514 records were identified through database searching, including Scopus (*n* = 152), PubMed (*n* = 267), and Web of Science (*n* = 95). After removing 157 duplicate records, 357 unique records remained for title and abstract screening. Of these, 314 records were excluded as they did not meet the eligibility criteria. Subsequently, 43 reports were sought for full-text retrieval; all were obtained and assessed for eligibility. Following full-text evaluation, 26 reports were excluded for the following reasons: not an RCT (*n* = 6), wrong population (*n* = 4), wrong outcome (*n* = 12), wrong intervention (*n* = 1), multi-component intervention (*n* = 1), intervention unclear (*n* = 1) and retracted article (*n* = 1). Finally, 17 studies met the inclusion criteria and were included in the review. The study selection process is presented in Figure 1.

### 3.2. Characteristics of the Included Studies

The 17 included RCTs were synthesised according to four broad categories: study and participant characteristics, intervention and comparator characteristics, psychological outcomes, and key findings. The results are presented below to describe the characteristics of the included studies, the nature and delivery of the MBIs, the outcomes assessed, and the patterns of findings reported across the included trials. The characteristics of the included studies are presented in Table 3, while the intervention characteristics are summarised in Table 4.

The publication years of the included studies indicate a growing research interest in MBIs among athletes over the past decade. The earliest included study was published in 2016 [78], followed by studies in 2019 [69,80,82,85], which together represent the highest number of publications across the included years. A gradual increase in publications was observed from 2020 onwards, including studies published in 2020 [74], 2021 [81], 2022 [75], 2023 [71,77], and 2024 [79,83]. Two studies were published in 2025 [70,73], and 3 studies were published in 2026 [72,76,84]. This trend suggests an increasing recognition of the importance of MBIs in sport psychology and athlete well-being research.

### 3.3. Geographical Location of the Studies

**Figure 2:**
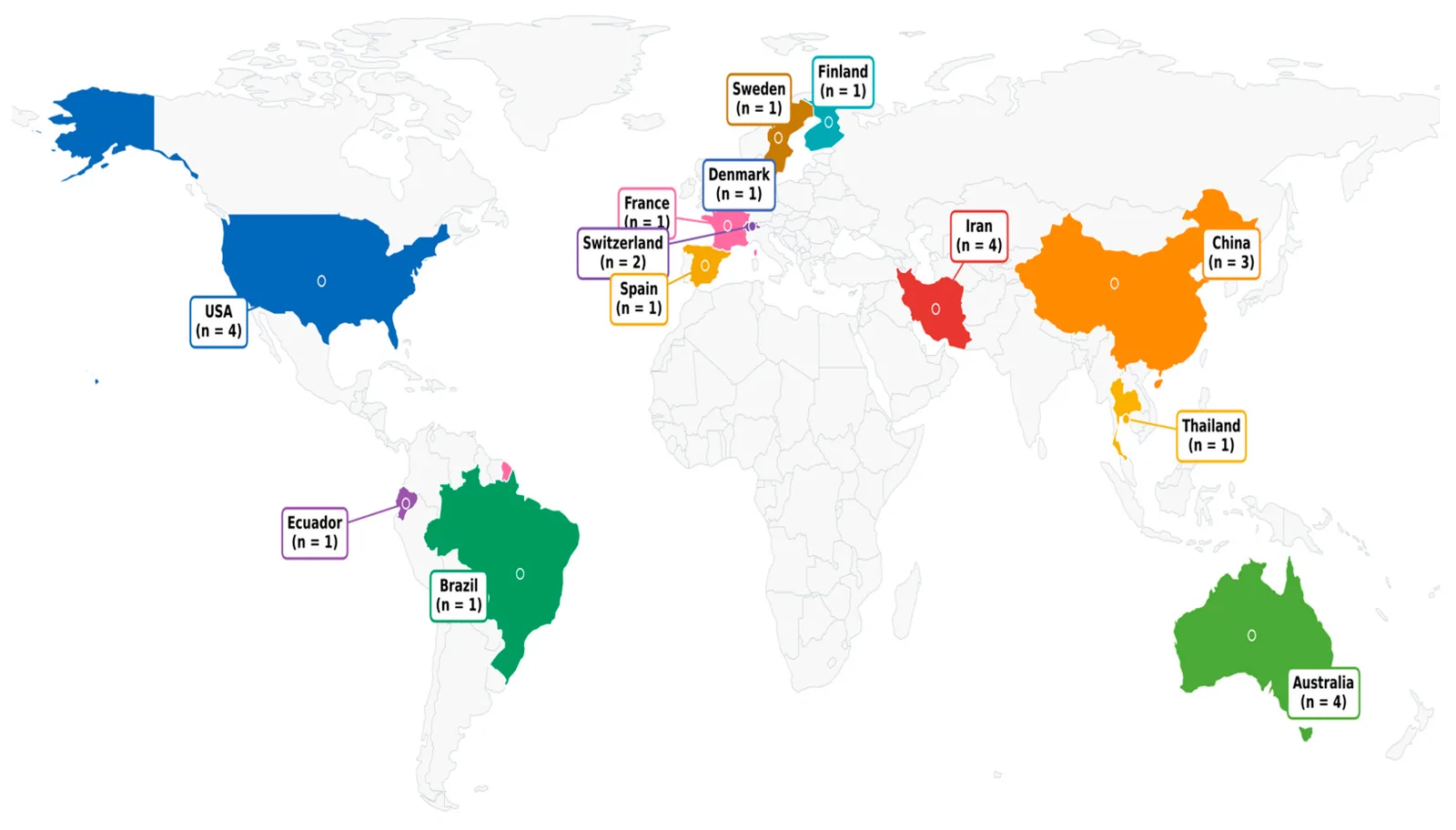
Geographical distribution of the 17 studies. Note: Country counts represent the countries associated with the included studies based on author affiliations. Where an article involved more than one country, each country was counted separately. Source: Prepared by the authors based on data extracted from the included studies.

The included studies were conducted across several geographical regions, indicating growing international interest in MBIs among athletes; however, the evidence was predominantly derived from high-income countries, with limited representation from middle-income settings. Among the 17 included studies, the highest country representation was observed for Iran [69,75,77,81], the USA [71,81,82,83], and Australia [69,75,78,85], with four studies each. This was followed by China [76,79,83], with three studies, and Switzerland [74,81], with two studies. Thailand [70], Finland [72], France [73], Brazil [77], Sweden [80], Denmark [84], Spain [84] and Ecuador [84] were each represented by one study. This geographical diversity indicates that MBIs in sport settings have been investigated across 13 countries spanning Asia, Europe, North America, South America, and Oceania, providing broader geographical and cultural representation of the evidence base. The geographical distribution of the included studies is presented in Figure 2.

This was followed by China, with three studies, and Switzerland, with two studies. Thailand, Finland, France, Brazil, Sweden, Spain, Denmark, and Ecuador were each represented by one study. Overall, the included studies represented 13 countries across Asia, Europe, North America, South America, and Oceania.

### 3.4. Participant Characteristics

The included studies demonstrated considerable diversity in participant characteristics, encompassing athletes from different sports, competitive standards, age groups, and demographic backgrounds. The 17 included RCTs had individual study sample sizes ranging from 18 to 288 participants, with a median sample size of 47 participants per study. Sixteen of the 17 included studies (94.1%) recruited fewer than 100 participants [69,70,71,72,73,74,75,76,77,78,79,80,81,82,84,85], indicating that most trials were based on relatively small samples. The smallest study included 18 elite Paralympic athletes [85], whereas the largest enrolled 288 college athletes [83]. These relatively small sample sizes may limit statistical power and the generalisability of findings across different athlete populations. The participants represented a wide range of athletic populations, including amateur athletes [69], collegiate and university athletes [70,71,76,82,83], national-level athletes [72,75], competitive and club-level athletes [73,74,78], elite athletes [77,80,81,84], and Paralympic athletes [85], thereby providing a diverse evidence base for evaluating the effectiveness of MBIs in sport settings.

Regarding sex distribution, the included studies comprised both male and female athletes, although notable variability was observed across studies. Two studies exclusively recruited female athletes [75,81], whereas one study included only male participants [69]. The remaining studies involved mixed-sex samples. Based on studies reporting sex-specific data, sex information was not explicitly reported in two studies [76,77]. Several studies had a predominance of female participants, with the proportion of female participants ranging from 60% in Ronkainen et al. [72] to 72% in Macdougall et al. [85] and 85% in Glass et al. [82]. In contrast, male athletes predominated in studies involving cyclists [78], handball players [84], and college athletes [83]. The age of participants varied considerably across the included studies. Mean participant age ranged from 19.32 years among collegiate athletes [82] to 41.7 years among competitive runners [73]. Most studies involved young adult athletes, with reported mean ages generally ranging from approximately 19 to 25 years. Two studies included relatively older athletic populations, namely competitive cyclists [78] and trained runners [73]. Athletes participating in Paralympic sports also demonstrated a higher average age [85].

The included studies encompassed a diverse range of sporting disciplines and athlete populations, reflecting the broad application of MBIs across competitive sport settings. Participants were drawn from both individual and team sports. Individual sports represented in the included studies included athletics and sprinting [70,72,73,79], cycling [78,80], swimming [70,82,83], badminton [70,74,83], table tennis [75,76,83], tennis [74,82,83], golf [80,83], fencing [83], taekwondo [83], wrestling [80], and Paralympic sports [85]. Team sports included basketball [69,72,83], football/soccer [70,80,82], beach soccer [77], volleyball [82,83], handball [84], floorball [74,80], lacrosse [82], field hockey [82], and cheerleading [83]. Several studies focused on athletes from a single sport [69,73,78,79,84], whereas others recruited participants from multiple sporting disciplines.

### 3.5. Study Design

Regarding study design, all included studies employed RCTs, consistent with the review’s inclusion criteria. The most common design was the parallel-group RCT, which was used in the majority of studies [69,71,73,75,77,80,81,82,83]. Several studies incorporated additional methodological features such as pre-test and post-test assessments [69,75], follow-up evaluations [75,77,81], and wait-list control conditions [78,82]. Variations in trial structure were also evident across the included studies. Three-arm RCTs were employed in two studies [70,74], allowing comparisons between MBIs, alternative psychological interventions, and control conditions. One study adopted a randomised controlled superiority design [72], while another utilised a single-blind randomised crossover design [76]. A single-blind RCT with a 2 × 3 mixed design was reported by Yu et al. [79], and a randomised pre-test–post-test training study was conducted among elite handball players [84]. Additionally, one study was designed as a pilot RCT with concealed allocation [85].

### 3.6. Intervention Characteristics

The included studies employed a wide variety of MBIs adapted specifically for sport and athletic settings. The most commonly implemented interventions were MSPE [69,71,82] and MAC-based interventions [75,77,80,81,85]. Other approaches included sport-specific ACT [72], mindfulness meditation training [70], brief mindfulness meditation programmes [73], Sport-specific mindfulness training based on mindfulness-integrated cognitive behavioural therapy (MiCBT) with a mindful spin-bike component [78], smartphone-delivered MBSR [83], Mindfulness Acceptance Insight Commitment (MAIC) training [79], Mindfulness Intervention and training [74,76], and hybrid mindfulness interventions using mPEAK and Headspace platforms [84]. Thus, the interventions varied not only in their theoretical and practical approaches but also in the extent to which they were manualized and sport-specific.

Considerable variation was observed in intervention duration, session frequency, and total intervention dose. Intervention durations can be seen up to 16 weeks [76], although most interventions were conducted between 6 and 8 weeks [69,70,71,72,73,75,77,78,79,80,81,82,83,84,85]. Session frequency also varied substantially. Several interventions were delivered once weekly [71,72,75,77,79,80,81,82,85], whereas others involved more intensive schedules, such as three sessions per week [70,73], four sessions per week [76], or daily mindfulness practice [78,83,84]. Session duration ranged from brief 5–12 min mindfulness practices [73] to 90 min group workshops [69,74].

The mode of intervention delivery also differed considerably among the included studies. Most interventions were delivered through face-to-face group sessions [69,71,72,74,75,78,80,81,82], while some incorporated individual-based approaches [85]. The smartphone-delivered MBSR intervention was delivered through the WeChat platform with therapist interaction [83]. In contrast, the mPEAK/Headspace intervention employed a hybrid delivery model that combined structured training sessions with guided digital mindfulness practices [84]. Several interventions also incorporated home-based mindfulness practice supported through audio recordings, mobile applications, guided CDs, or emailed mindfulness exercises [69,73,75,78].

Adherence, therapist contact, home practice, and intervention fidelity were also considered during data extraction; however, these characteristics were not reported consistently across the included trials. Where reported, adherence was described in terms of participation, practice completion, or engagement with the intervention rather than inferred from intervention outcomes. Information concerning therapist involvement and home practice also varied across studies, while formal assessments of intervention fidelity were limited. Consequently, these characteristics were treated as descriptive intervention features and were not used to explain differences in intervention effects unless the original study directly examined or reported such associations. The substantial variation in delivery formats and intervention intensity also limited direct comparisons of individual intervention components.

The comparator conditions varied across studies and included both passive and active control groups. Passive controls commonly involved usual coaching, wait-list controls, usual activities, or no/minimal intervention [69,72,75,76,77,78,82,85]. In contrast, active comparator conditions included psychological skills training (PST) [71,74,80], imagery training [70], running information combined with one progressive muscle relaxation session [73], sport psychology educational programmes [81], psychological guidance interventions [79], mental health education materials [83], and an active control condition [84]. The inclusion of active control groups allowed comparisons between MBIs and alternative psychological or behavioural interventions.

The included interventions targeted a broad spectrum of psychological and performance-related outcomes. Frequently assessed constructs included mindfulness [69,73,75,78,79,80,81,82,83,84], anxiety [70,71,78,79,82,83], psychological well-being [72,75,85], emotional regulation [71,74,80], mental toughness [69,75], flow state [71,78,82], self-compassion and grit [81], cognitive functioning [76,84], rumination and cognitive flexibility [77], and sport performance outcomes [71,77,80,82,84]. Findings varied across outcomes and interventions, with improvements reported for selected mindfulness, emotional regulation, mental well-being, cognitive, stress-related, and sport-related outcomes [69,72,75,76,77,78,80,81,82,84,85]. However, mixed or non-significant findings were also reported for anxiety, self-esteem, acceptance, depression, and other outcomes [70,72,73,78,83]. These findings indicate heterogeneity in reported outcomes rather than a uniform pattern of benefit across interventions.

### 3.7. Key Findings

The included studies reported heterogeneous findings across psychological well-being, emotional regulation, cognitive functioning, and sport-related psychological outcomes. Improvements in mindfulness-related constructs were reported across several interventions, although the direction and extent of findings varied across studies and comparator conditions [69,73,75,78,79,80,81,82]. For example, MSPE was associated with improvements in mindfulness, mental toughness, and emotional intelligence among amateur basketball players [69]. MAC interventions were also associated with improvements in mindfulness, mental toughness, psychological well-being, self-compassion, and grit among national-level and elite athletes [75,81]. In addition, one study reported greater improvements in mindfulness, perceived performance, and emotional regulation following MAC compared with PST [80].

Findings for anxiety, stress, and emotional regulation varied across interventions and outcome measures. Mindfulness meditation training reduced cognitive state anxiety among university athletes [70], while a sport-specific ACT intervention improved mental well-being and reduced stress symptoms among national-level athletes [72]. Similarly, a MiCBT intervention was associated with improvements in mindfulness and flow experiences and reductions in pessimistic attribution styles among competitive cyclists [78]. Improvements in emotional regulation and attentional control were reported following both mindfulness training and PST, while mindfulness training additionally reduced experiential avoidance [74].

Several studies reported improvements in cognitive and sport-related psychological outcomes. Mindfulness intervention was associated with improvements in cognitive functioning and emotional state, reduced mental fatigue, and lower cortisol levels among college athletes [76]. Among elite beach soccer players, an MBI was associated with improved cognitive flexibility, reduced rumination, and enhanced sport performance [77]. The hybrid mPEAK and Headspace intervention was associated with improved cognitive performance and response inhibition, reduced mental fatigue, demand, and frustration, and increased dispositional mindfulness among elite athletes [84]. Other reported outcomes included flow state, life satisfaction, sport anxiety and enjoyment, performance satisfaction, worry, and perceived athletic performance [71,78,80,82,85].

Despite the predominantly positive findings, some studies reported mixed or non-significant effects for specific psychological outcomes. For example, mindfulness meditation training did not produce significant improvements in somatic anxiety, self-confidence, or HRV despite reductions in cognitive anxiety [70]. The sport-specific ACT intervention did not significantly improve depression symptoms, self-esteem, or recovery experiences [72]. The BMM intervention improved refocusing ability and selected well-being outcomes but did not significantly affect acceptance [73]. Similarly, the smartphone-delivered MBSR programme did not significantly improve anxiety, although a near-significant improvement was observed for the mindfulness observation facet [83]. Poor adherence related to academic pressures and time constraints was identified as a potential factor contributing to these limited findings [83]. Several studies also demonstrated sustained intervention benefits during follow-up assessments. Improvements in mindfulness, mental toughness, and psychological well-being following MAC were maintained at two-month follow-up [75], while gains in self-compassion and grit remained stable at four-week follow-up [81]. Treatment completers in the MSPE intervention also demonstrated maintained improvements in flow, life satisfaction, and mindfulness, together with reductions in worry, at five- to six-month follow-up [82].

## 4. Discussion

This scoping review synthesised empirical evidence from RCTs examining MBIs and psychological outcomes among athletes. By focusing exclusively on RCTs, the review provides a focused map of the available experimental evidence while recognising substantial heterogeneity in intervention approaches, outcome measures, comparator conditions, and athlete populations. The discussion is structured around six research questions: (1) how MBIs are conceptually defined and described in RCTs involving athletes; (2) how MBIs are operationalised, including intervention characteristics and delivery approaches; (3) which psychological well-being outcomes have been examined and what patterns of findings have been reported; (4) what patterns of psychological outcomes have been reported when MBIs are compared with active control conditions; (5) what intervention characteristics have been reported and what patterns of outcomes are observed across these characteristics; and (6) how sport-adapted and standardized MBI approaches have been implemented and what psychological outcomes have been reported.

Research Question 1: How are MBIs conceptually defined and described in RCTs involving athletes?

The 17 included RCTs reflected several distinct theoretical and intervention traditions within mindfulness-based sport research. The most commonly used protocol [69,71,82], MSPE, was specifically designed for the athletic population and progresses from sedentary to dynamic exercise and finally to sport-specific applications. Five studies used the MAC approach [75,77,80,81,85], which is rooted in ACT and a values-based, acceptance-oriented paradigm of mindfulness, but applied to high-performance sport contexts. Four of these studies employed non-manualised mindfulness meditation training programmes [70,73,74,76], one an ACT sports-based programme [72], another one is MAIC [79], one a MiCBT programme with a mindful spin-bike element [78], and one a hybrid digital platform that delivered MBSR [83] and one commercial mindfulness application (mPEAK and Headspace) summed [84].

Variability in intervention type makes cross-study synthesis difficult. MBIs differ not only in their names but also in their theoretical foundations, key mechanisms, training focus, and delivery models. MSPE focuses on attentional regulation in the here and now and on integrating mindfulness while executing sport-specific skills [69,71,82]. Psychological flexibility, values clarification, and experiential acceptance are preconditions for performance and well-being, according to MAC [75,77,80,81,85]. In the original clinical formulation of MBSR, the focus is on non-judgmental awareness and on stress reduction through structured formal practices. These differences suggest that the interventions may involve different active components and mechanisms of change, although the included RCTs did not permit direct comparison of these mechanisms. The phrasing ‘MBI’ (as used in many previous reviews) can be misleading in that it obscures what actually helps, whom, and when.

One of the more interesting findings concerns the two MBI trials delivered digitally, identified in this review [83,84]. Poor adherence was observed in the smartphone-delivered MBSR study [83] (53.4% adherence rate), which showed no significant effect on primary anxiety outcomes, likely due to the scheduling pressure of participants’ academic and training activities. Likewise, the mPEAK/Headspace intervention [84] had a weak effect, as low uptake reduced its impact. The findings point to an important hurdle in the delivery of digital MBI to athletes: time constraints that can make digital delivery desirable also make it difficult when engagement depends on persistent daily effort. Future research could investigate whether shorter, contextually embedded mindfulness practices are feasible for athletes with demanding training and academic schedules and whether such approaches can improve adherence and intervention engagement.

Research Question 2: How are MBIs operationalised in RCTs, including their intervention type, duration, frequency, session length, delivery format, and instructor characteristics?

### 4.1. Operationalisation of Mindfulness-Based Interventions

Across the included RCTs, MBIs were operationalised through varied intervention types, durations, frequencies, session lengths, delivery formats, and home-practice requirements. Although standardised measures were commonly used, substantial heterogeneity in outcome selection, reliance on self-reported measures, and limited incorporation of objective indicators were also evident.

#### 4.1.1. Outcome Measures

The included studies assessed outcomes using a range of established self-report, cognitive, psychophysiological, and performance-based measures. Although many of the instruments have established psychometric properties, substantial heterogeneity was observed in their conceptual focus and measurement characteristics.

##### Psychological Distress and Emotional States

Psychological distress, anxiety, stress, and emotion regulation were assessed using different instruments across the included studies, including the Depression Anxiety Stress Scales-21 (DASS-21), Sport Anxiety Scale-2 (SAS-2), Competitive State Anxiety Inventory-2R (CSAI-2R), Perceived Stress Scale-10 (PSS-10), and Difficulties in Emotion Regulation Scale (DERS) [70,71,72,78,82]. These measures primarily capture symptoms of distress, anxiety, perceived stress, and emotion regulation rather than positive psychological well-being. Their use therefore reflects the breadth of psychological outcomes assessed across the included RCTs rather than a single conceptual construct.

##### Positive Psychological Functioning and Well-Being

Positive psychological functioning and well-being were assessed using measures including the Emotional Intelligence Scale [69], Self-Regulation Form and Positive Experience Questionnaire (PEQ) [71], Resilience Evaluation Questionnaire (REQ), Perceived Functional Social Support Scale (PFSS), and Mental Health Continuum-Short Form (MHC-SF) [72], Subjective Happiness Scale (SHS) [76], Ryff’s Psychological Well-Being Scale [75,85], Satisfaction with Life Scale (SWLS) [82,85], Scale of Positive and Negative Experience (SPANE) [85], Cognitive and Affective Mindfulness Scale-Revised (CAMS-R) [81], Spiritual Well-Being Questionnaire (SWBQ) [85], Self-Compassion Scale-Short Form (SCS-SF) [81], Difficulties in Emotion Regulation Scale (DERS) [80] Short Grit Scale (Grit-S/SG-S) [81], Other measures include the Cognitive Flexibility Inventory (CFI) [77], Social Adaptation Self-Evaluation Scale (SASS) [78] and Rosenberg Self-Esteem Scale (RSES) [72]. These measures encompass overlapping aspects of positive functioning, affect, psychological resources, and well-being rather than representing a single homogeneous outcome domain.

##### Mindfulness and Cognitive-Affective Mechanisms

Theoretically relevant measures of mechanism that are thought to be central processes in the actions of MBIs, such as the Five Facets Mindfulness Questionnaire (FFMQ) [76,78,82,83], the Philadelphia Mindfulness Scale (PHLMS) [71], the Mindfulness Inventory for Sport (MIS) [71], Mindful Attention Awareness Scale (MAAS) [84], Acceptance and Action Questionnaire-II (AAQ II) [82] and the Athlete Mindfulness Questionnaire (AMQ) [72], measured experiential avoidance and non-reactive observation, cognitive flexibility, and mindfulness, respectively.

##### Sport-Specific Psychological Outcomes and Performance Indicators

Sport-related psychological and performance indicators included the Mindful Sport Performance Questionnaire [69], the Sport Mental Toughness Questionnaire (SMTQ) [75], Flow State Scales, Dispositional Flow Scale-2 (SDFS-2) [71], Flow State Scale-2 (CDFS-2) [71], and Rating of Perceived Exertion (RPE) [76]. These measures complemented broader psychological outcomes by assessing sport-specific psychological functioning and performance-related characteristics.

One of the cross-cutting concerns addressed was the widespread use of self-report measurement. Podsakoff et al. have shown that when outcomes are solely self-reported, common method variance, social desirability bias, and demand characteristics pose threats to the construct validity of the outcomes, especially when they are administered by coaches or sport psychologists known to the participants [86]. Only one of the studies reviewed incorporated objective psychophysiological indices (cortisol levels and prefrontal oxygenation) in addition to subjective self-report measures [76], and a smaller number of trials incorporated objective or performance-based measures alongside self-report outcomes. Qian et al. included cognitive tasks and psychophysiological measures, including salivary cortisol and prefrontal oxygenation, while Staiano et al. incorporated the Sustained Attention to Response Task (SART) and Stroop task to assess cognitive performance [76,84]. Future RCTs would greatly benefit from the systematic incorporation of objective biomarkers, wearable sensor data, and neurocognitive assessments alongside self-reported outcomes.

##### Analytical Approaches

One of the strengths of the RCT design is its ability to distinguish intervention-related changes from changes occurring in control conditions. Across the 17 included trials, this strength was addressed through the use of both passive and active comparator conditions. Most studies used passive control groups, such as individuals who received the standard level of coaching treatment with no additional psychological input, or, in some studies, informational materials (e.g., relaxation brochures, general health pamphlets) [69,70,72,74,75,76,77,78,82,85]. Comparisons with passive controls may reflect both intervention-specific and non-specific effects, including attention, expectancy, group support, and participant engagement.

Nine studies included active comparator conditions [70,71,73,74,79,80,81,83,84]. These included imagery training [70], PST [71,74,80], a control condition receiving the same running programme with an additional progressive muscle relaxation component [73], psychological guidance [79], sport psychology education [81], mental health education materials [83], and an active control condition in the mPEAK/Headspace trial [84]. In the latter study, mPEAK/Headspace was the mindfulness intervention rather than the comparator. Direct comparisons between MBIs and PST were reported in three trials [71,74,80]. Hut et al. [71] found improvements in sport anxiety and enjoyment in both groups, with greater improvement in satisfaction with sport performance in the MSPE group. Röthlin et al. [74] reported improvements in emotion regulation and attention-related outcomes following both mindfulness training and PST, while mindfulness training additionally reduced experiential avoidance. Josefsson et al. [80] reported greater improvements in dispositional athletic mindfulness, emotional regulation, and perceived training performance following MAC compared with PST.

Other active-comparator trials produced outcome-specific findings. Mindfulness meditation training reduced cognitive anxiety compared with imagery training, but did not significantly affect somatic anxiety or self-confidence [70]. The BMM intervention improved selected mindfulness and well-being outcomes compared with its active control condition [73], while MAC was associated with greater improvements in mindfulness, self-compassion, and grit than sport psychology education [81]. The smartphone-delivered MBSR intervention did not significantly improve anxiety outcomes compared with mental health education materials [83]. The mPEAK/Headspace intervention was associated with improvements in cognitive performance, dispositional mindfulness, and reductions in mental demand and fatigue relative to its active control condition [84].

The direct head-to-head evidence suggests that MBIs and PST may produce overlapping benefits for some psychological outcomes, while MBIs may provide additional benefits for selected mindfulness-related or mechanism-specific outcomes. However, these findings are outcome-specific and do not establish general equivalence or superiority of MBIs over PST or other active interventions. Differences observed across studies using different comparator conditions should be interpreted as indirect cross-study observations rather than direct evidence of comparative effectiveness. The heterogeneity of comparator conditions remains an important methodological consideration and highlights the need for future RCTs using theoretically matched active controls that are comparable in intervention duration, contact time, attention, and expectancy.

Research Question 3: What psychological well-being outcomes have been examined, and what patterns of findings have been reported regarding the effects of MBIs among athletes?

### 4.2. Conceptualisation of Psychological Well-Being

The findings of the present review are broadly consistent with, while also extending, previous evidence syntheses of MBIs in athletic populations. Noetel et al. [62] reported beneficial effects on mindfulness, flow, athletic performance, and competitive anxiety, although the evidence was rated low quality and strong causal conclusions were discouraged. Similarly, Bühlmayer et al. [36] found beneficial effects on mindfulness and psychological performance surrogates, while performance effects were limited to precision sports. Myall et al. [65] reported improvements in mental health, including anxiety, stress, and psychological well-being, although substantial heterogeneity was observed. Wang et al. [1] found significant effects on mindfulness and mindfulness-related psychological components, but not on overall mental health, with substantial heterogeneity. Ptáček et al. [87] reported moderate effects on mindfulness and small effects on sport performance, but insufficient evidence to support MAC for improving well-being. The findings indicate potentially beneficial effects across selected psychological outcomes, although the direction and magnitude of effects varied substantially across studies.

The present review identified reported improvements across several psychological well-being outcomes, although findings varied substantially by intervention type, outcome, comparator, and athlete population. Taken together, these findings suggest that although MBIs show promising effects across several psychological domains, their effects are not uniform and may vary according to the specific psychological construct, intervention approach, comparator condition, athlete population, and measurement strategy. In contrast to previous broader evidence syntheses, the present review specifically maps RCT evidence concerning psychological well-being among athletes, providing a more focused characterisation of intervention and outcome heterogeneity and identifying areas requiring further investigation. Although non-randomised studies may provide complementary information on real-world implementation, the present review focused specifically on RCT evidence in accordance with its predefined eligibility criteria.

In the 17 RCTs included in this review, psychological well-being is clearly a multi-dimensional construct; the variables in these studies have been operationalised across a wide range of dimensions, corresponding to the multifaceted nature of athlete health and the theoretical diversity of the field. Studies found reported outcomes included trait and state mindfulness, mental toughness, emotional intelligence, cognitive anxiety, competition state anxiety, mental well-being, emotional regulation, attention, experiential avoidance, psychological flexibility, mental toughness, burnout, self-compassion, grit, cognitive performance, mental fatigue, demand, emotional state, sport-related rumination, athletic coping skills, flow states, self-efficacy, satisfaction with life, cognitive flexibility and eudaimonic indices such as positive relations with others, personal growth, and sense of purpose [69,70,72,73,74,75,76,77,78,79,80,81,82,84,85]. This is actually a strength and a complication at the same time.

What was compelling about the studies was their ecological validity: they reflected, to varying degrees, the lived psychological reality of athletes throughout training and competition, rather than focusing solely on the symptom cluster of well-being. However, this diversity also exposes a constant conceptual confusion. Research on psychological well-being in athletes has traditionally been operationalised in two epistemologically distinct ways: Hedonic well-being, which focuses mainly on positive affect, life satisfaction, and lack of distress, and Eudaimonic well-being, which emphasises meaning, personal growth, and psychological functioning [88]. Many of the included RCTs focused on the deficit-reduction end of the spectrum, with most emphasising reductions in anxiety, depression, and stress [70,71,72,78,79,83]. In contrast, fewer RCTs systematically evaluated positive psychological outcomes, self-compassion, and/or eudaimonic aspects [73,75,77,81,82,85].

This skew is clinically comprehensible, as psychological stress during performance is acute, but it may offer only a partial view of what it means to be a successful athlete. Rather than viewing athlete well-being as simply the absence of psychological disorders, Gross [2] describes it as the presence of adaptive psychological strengths, including resilience, equanimity, self-awareness, and purposeful engagement, which promote consistent performance and support a healthy identity beyond sport. It would be beneficial for both the hedonic and eudaimonic aspects of the conceptual framework to be present, creating a more comprehensive evidence base for sport psychology practitioners to draw on to inform more meaningful practice.

Research Question 4: What patterns of psychological outcomes have been reported when MBIs are compared with active control conditions in RCTs involving athletes?

The comparative efficacy question is perhaps most practically salient in the light of this review, and the nine studies among the 17 RCTs that included an active comparator condition provide valuable, though qualified, insights. The available comparisons with active control conditions showed outcome-specific patterns, with some studies reporting additional benefits for MBIs on selected psychological outcomes. One of the most methodologically sound trials included in this review, by Röthlin et al., compared Mindfulness Training and PST with a passive wait-list control, involving 95 athletes from four Swiss sport federations [74]. Mindfulness Training and PST showed significant improvements in emotional regulation and attentional control awareness compared to the wait-list condition, which, as mentioned, is a well-established benefit of structured psychological training. However, critically, Mindfulness Training (and Mindfulness Training alone) was shown to significantly decrease experiential avoidance, or the suppression, avoidance, or escape from unwanted internal experiences. This finding has a significant theoretical contribution: experiential avoidance is a central process that associates anxiety and rumination with decrements in performance and burnout in athletes [70,71,74,77]. The reduction in experiential avoidance observed following Mindfulness Training may indicate a potential psychological process associated with the intervention’s effects; however, this interpretation requires further investigation in studies specifically designed to examine underlying mechanisms.

In a study in Sweden, Josefsson et al. compared the MAC with structurally similar PST in 69 elite athletes [80]. The results showed that dispositional mindfulness of athletes in the sport domain was higher post-intervention in the MAC group, and that self-rated athletic performance was higher in the MAC group, with a statistically significant mediation between dispositional mindfulness and self-rated athletic performance in the group. Results indicated that sport-specific dispositional mindfulness, emotion regulation, and self-rated athletic performance increased post-intervention in the MAC group, and that both dispositional mindfulness and emotion regulation were statistically significant mediators between MAC and self-rated athletic performance. This mediation finding is noteworthy because it provides preliminary evidence of an association between changes in dispositional mindfulness, emotion regulation, and self-rated athletic performance. However, these findings should not be interpreted as establishing a causal mechanism, and further studies specifically designed to examine mediating pathways are warranted.

The same pattern was found by Mohebi et al., who compared the effects of MAC with an active sport psychology education control in elite female Iranian athletes, with significantly greater improvements in self-compassion and grit for the MAC group and stable effects at 4-week follow-up [81]. The psychological attributes outlined above are adaptive strengths that may be among the most enduring and clinically significant outcomes of MAC-based interventions, as they have previously been linked to athlete resilience and recovery from setbacks [70,73], a link that is not captured by typical deficit-oriented outcome measures.

The picture is more complicated for anxiety in particular. Pakulanon and Petviset [70] reported that they achieved a magnitude of change in cognitive state anxiety but not in somatic anxiety or self-confidence as a result of mindfulness meditation training compared to an imagery training comparator. Gao et al. [83] found no significant difference in anxiety between the smartphone-delivered MBSR and mental health promotion control groups. Although adherence was relatively low, this may have influenced the observed findings; however, the null result may also reflect limited efficacy of the intervention in this population. Yu et al. found that there were meaningful differences between the competition state anxiety scores as compared to a psychological guidance control group [79]. The available findings suggest that reductions in cognitive anxiety may be more consistently reported than reductions in somatic anxiety; however, this pattern should be interpreted cautiously because the relevant trials differed in intervention and comparator characteristics. This pattern is theoretically consistent with the attentional retraining mechanisms that underpin mindfulness and is congruent with the meta-analysis results that showed moderate to large effects of MBIs on cognitive anxiety in sport [35,45].

The overall meta-analysis literature confirms these patterns. In a systematic review and meta-analysis of mindfulness-based programs for elite athletes’ mental health, Myall et al. [65] identified significant reductions in anxiety and stress, as well as overall improvements in mental health. Wang et al. [1] found significant effects for mindfulness and related psychological components, but no significant overall effect on mental health. Effects on athletic performance were described narratively rather than through meta-analysis. Specifically, the findings from the within-review RCTs were similar to those of Ptáček et al. [87], showing large effects of MAC on mindfulness and small yet positive effects on athletic performance. The findings suggest that selected sport-adapted approaches may provide benefits for specific psychological outcomes relative to some active comparators; however, the heterogeneity of interventions and outcomes prevents conclusions regarding the comparative effectiveness of MBIs as a unified category.

Research question 5: What intervention characteristics, including duration, frequency, and session length, have been reported in RCTs examining MBIs among athletes, and what patterns in psychological outcomes are observed across these characteristics?

The included RCTs varied considerably in intervention duration, session frequency, and session length, allowing these characteristics to be mapped descriptively but not compared as experimentally manipulated dose parameters. The RCTs included ranged in duration from 4 weeks [74], 6 weeks [69,71,72,82,83,84], 7 weeks [75,77,79,80,81], 8 weeks [70,73,78,85], and 16 weeks [76]. Röthlin et al. [74] reported positive findings for an MSPE protocol with four 90 min group sessions and a highly structured comparator design. In contrast, most studies (15/17) used interventions of moderate length (six to eight weeks). Most studies used interventions lasting six to eight weeks, with beneficial findings reported across several psychological domains; however, the available evidence does not permit determination that this duration is superior to shorter or longer interventions [69,70,71,72,73,75,77,78,79,80,81,82,83,84,85]. The one study that lasted longer than 8 weeks (16-week mindfulness training for Chinese table tennis players) showed beneficial effects on cognition, emotional regulation, and mental fatigue as well as objective biomarker changes, which may indicate that longer duration programmes may be better at inducing neurocognitive and psychophysiological changes that shorter programmes are not [76].

The duration of sessions in the studies included ranged from 5 to 12 min of mindfulness meditation [73] to 90 min of sessions in the MSPE-based protocol [69,74]. Protocols that are six to eight weeks long are delivered either once or twice weekly, typically with structured home practice, guided audio recordings, mobile applications, or exercises sent via email [69,70,71,72,73,75,77,78,79,80,81,82,83,84,85]. Several MSPE and MAC trials have included additional guided imagery and guided activities at home that were reported to help reduce symptoms, especially anxiety, by building the skills of attention and self-regulation taught in the group sessions [69,71,75,80,81,82,85]. This finding supports observations from studies examining the effects of home practice engagement on MBIs’ outcomes in clinical populations, although this relationship has not yet been formally tested in RCTs involving athletes [89,90,91].

Overall, the included studies provide a descriptive profile of commonly used intervention durations, frequencies, and home-practice approaches; however, these patterns should not be interpreted as evidence of an optimal intervention dose. Although several studies using 6–8-week protocols reported beneficial findings, no RCT systematically manipulated dose parameters within the same study; therefore, an optimal dose cannot be established from the available evidence. No RCT found used a systematic manipulation of dose parameters within a single study, which prevented the separation of duration effects from the possibility that other aspects of the intervention (type, delivery, or athlete characteristic) may have confounded the results. Future studies need to focus on factorial dose-finding RCTs to determine empirically based dosing recommendations for sport-specific implementation of MBI.

Research Question 6: How have sport-adapted and standardised MBI approaches been implemented in RCTs involving athletes, and what psychological outcomes have been reported?

### 4.3. Sport-Adapted and Standardised Approaches

The basic rationale for sport-specific adaptation is that the psychological demands imposed during athletic contests differ from those in clinical contexts, as the latter are not intended to address the unique demands of sport. There is a reasonable basis for the current evidence to support this premise.

#### 4.3.1. Mindfulness-Acceptance-Commitment-Based Interventions

The five RCTs using MAC-based protocols reported improvements across several psychological outcomes, although the specific outcomes and magnitude of reported benefits varied between studies [75,77,80,81,85]. Josefsson et al. showed that a structurally similar PST was less effective than MAC in sport-specific mindfulness, emotion regulation, and perceived athletic performance [80]. Mohebi et al. demonstrated a significant increase in self-compassion and grit resulting from the MAC that was much larger than the increase resulting from an active sport psychology education control [81]. Sabzevari et al. showed that MAC not only decreased rumination but also increased cognitive flexibility and sports performance in elite beach soccer players, even at a two-month follow-up [77]. Macdougall et al. extended this evidence to Paralympic athletes, and they found that overall life satisfaction, personal growth, positive perceptions of relationships with others, and pain perceptions improved significantly in sport MBI research [85]. Ajilchi et al. showed significant improvements in mindfulness, mental toughness, and psychological well-being [75].

In a sport-specific ACT study, Ronkainen et al. examined important nuances among athletes at the national level in athletics and basketball in Finland [72]. While this trial showed large effects on stress reduction and psychological flexibility, no significant effects on depression, recovery experience, or self-esteem were observed, which may be due to selective effects on well-being (specifically, the mechanisms targeted by ACT values-based action and psychological flexibility) rather than to symptom reduction.

#### 4.3.2. Mindful Sport Performance Enhancement-Based Interventions

The most explicitly sport-designed protocol reviewed herein was MSPE [69,71,82]. In a methodologically sound parallel RCT involving 52 NCAA Division III athletes, Glass et al. reported significant improvements in flow, trait mindfulness, life satisfaction, and self-rated sport performance, as well as decreased worry, at a 6-month follow-up after the intervention [82]. The present longitudinal outcome is especially remarkable given the literature’s focus on the immediate post-intervention period. It is consistent with data from Thompson et al., who reported long-term benefits of MSPE on flow and sport anxiety 1 year following the intervention [92].

After 7 weeks, Ajilchi et al. observed large effects in mindfulness, mental toughness, and emotional intelligence among the female athletes with MSPE [69]. Hut et al. found that both MSPE and PST positively affected sport anxiety and sport enjoyment, with the MSPE group showing higher satisfaction with sport performance, which directly impacts athletes’ quality of life and retention in sport [71]. In fact, an intensive six-day online delivery of MSPE was found to yield mixed outcomes, with improvements in athletic coping skills, confidence, and achievement motivation, but no significant effect on total mindfulness [93].

One of the secondary findings from the MSPE research was that there is some level of moderation: The data from Hut et al. indicated that athletes with lower expectations of learning mindfulness skills showed higher scores on the concentration scale and lower scores on psychological inflexibility compared to athletes with higher expectations of learning mindfulness skills [71]. This interaction between expectancy and outcome is clinically relevant and indicates that the framing and presentation of MSPE to athletes could have a meaningful impact on its psychological effects.

Four RCTs employed non-manualized mindfulness meditation training, demonstrating a pattern of domain-specific rather than general psychological changes. Qian et al. showed that 16 weeks of mindfulness training for table tennis athletes resulted in meaningful reductions in mental fatigue, emotional state, and cortisol, and increases in prefrontal oxygenation (rare RCT-level evidence of changes in psychophysiological parameters with mindfulness training in athletes) [76]. After eight weeks of mindfulness meditation training, Pakulanon and Petviset reported a selective decrease in cognitive, but not somatic anxiety [70]. Röthlin et al. found mindfulness training to be a unique treatment for reducing experiential avoidance compared to PST [74]. Even though there were only a few other psychological changes, Staiano et al. demonstrated that the mPEAK/Headspace protocol enhanced neurocognitive performance, evidenced by improvements in SART accuracy and Stroop reaction time, while also increasing dispositional mindfulness and reducing mental fatigue and perceived mental demand. However, its effects on other psychological outcomes were limited in elite handball players [84].

The single MBSR trial conducted with 288 college athletes reported no effects of the MBSR treatment on primary anxiety measures, and adherence was found to be the biggest barrier [83]. This finding highlights the importance of critically evaluating contextual adaptation, adherence, and engagement when considering clinical MBI protocols for athletes. Importantly, this trial found that baseline anxiety was higher and mindfulness was lower in team-sport athletes compared to individual-sport athletes, which has implications for the meaningful tailoring of MBI content and delivery to the sport type, if this finding is replicated in other trials [83]. Scott-Hamilton et al. used a sport-specific mindfulness training that incorporated a new mindful spin-bike component, resulting in improvements in mindful measures, global flow, and sport-related pessimistic attributions among competitive cyclists [78]. The novel element of developing a mindfulness intervention specifically for sports-related physical activity, rather than a standalone cognitive activity, is a promising avenue for future interventions, especially for athletes who may see sedentary mindfulness practices as incongruent with their sport identity.

The available evidence does not permit meaningful comparison of standardised and sport-adapted MBI approaches because only a limited number of trials examined standardised protocols and the interventions differed simultaneously in delivery mode, adherence, duration, and content. The null findings from smartphone-delivered MBSR therefore cannot be attributed to its standardised clinical origins, nor can the more favourable findings from MSPE and MAC be attributed solely to their sport-specific adaptation. Future head-to-head RCTs should directly compare standardised and sport-adapted approaches while controlling for intervention dose, delivery mode, contact time, adherence, and expectancy.

### 4.4. Practical Implications

The findings may inform future research on the implementation and optimisation of structured MBI programmes within athlete support settings, including programmes delivered by sport psychologists, mental performance consultants, and team medical staff. However, the available evidence is insufficient to support prescriptive recommendations regarding intervention dose or delivery format. First, the predominance of six- to eight-week protocols in the available RCTs provides a useful description of current intervention practice but should not be interpreted as evidence that this duration is optimal. Future adequately powered dose-ranging and comparative RCTs should examine the effects of intervention duration, session frequency, session length, and overall exposure on psychological well-being and determine whether specific dose characteristics are associated with sustained benefits. This is particularly important because no included RCT systematically manipulated dose parameters, limiting the ability to distinguish duration effects from other intervention characteristics. Second, home practice warrants further investigation as a potentially important component of MBI delivery. Although several included interventions incorporated guided home activities, the independent contribution of home practice to outcomes has not been directly established in athlete RCTs. Future studies should therefore examine whether structured audio-, app-, or exercise-based home practice improves adherence, engagement, and psychological outcomes compared with interventions without additional home practice.

Third, team-based delivery may represent a feasible implementation option within athletic environments because it can utilise existing team structures and opportunities for shared practice. However, the available evidence does not establish that team-based delivery produces greater benefits than individual or other delivery formats. Direct comparative trials should therefore examine the relative effectiveness, acceptability, adherence, and feasibility of team-based, individual, and hybrid approaches. The substantial variation in delivery formats across the included studies further supports the need for such comparative research. Fourth, the evidence regarding digital delivery remains limited and heterogeneous. One smartphone-delivered MBSR trial reported adherence as a major barrier, whereas another hybrid intervention combining structured training with guided digital mindfulness practices reported improvements in selected cognitive outcomes. Accordingly, the current evidence does not justify recommending either conventional digital platforms or redesigned digital interventions as superior approaches. Future research should compare standalone digital, therapist-supported digital, hybrid, and training-integrated approaches while examining adherence, engagement, feasibility, and psychological outcomes. Ecological momentary or brief mindfulness practices integrated into training may be a promising avenue for investigation, but their comparative effectiveness remains to be established. Finally, future implementation studies should systematically monitor adverse events, potential harms, acceptability, adherence, and intervention fidelity, as these factors were not consistently synthesised across the available RCT evidence.

### 4.5. Limitations and Future Directions

There are some limitations to this scoping review that should be taken into consideration when interpreting its findings. First, the restriction to RCTs may have reduced the breadth of evidence captured by excluding quasi-experimental and pre–post intervention studies. Although this criterion was adopted to focus the review on experimental evidence, such studies may provide complementary information regarding intervention feasibility, implementation, and preliminary effects in athletic settings. Second, the included RCTs were mostly conducted in high-income countries such as the United States, Australia, Finland, France, Switzerland, Sweden, and Denmark, although studies from middle-income countries, including Iran, China, and Thailand, were also represented. Nevertheless, the relatively limited representation of low- and middle-income countries and other underrepresented regions may limit the generalisability of the findings. Third, most included trials had relatively small sample sizes, ranging from 18 to 288 participants [83,85], which may limit the precision and generalisability of the findings. Fourth, the high degree of variation in the types of MBI and across the different delivery methods such as MSPE [69,71,82], MAC [74,75,80,81,85], mindfulness meditation training [70,73,74,76,84] and digital delivery formats [83,84], sport-specific ACT [72,78] and outcome measurement instruments limited direct comparability across studies. As this study was designed as a scoping review to map the available RCT evidence, a formal meta-analysis was not prespecified or undertaken. However, this heterogeneity does not preclude quantitative synthesis a priori, and future systematic reviews should evaluate the feasibility of pooling effects according to specific outcomes, intervention types, and comparator conditions. Only 4 of those trials included any follow-up outcome measures [75,77,81,82], leaving the longer-term durability of MBI effects insufficiently characterised.

A further limitation is that the literature search was restricted to Scopus, Web of Science, and PubMed; therefore, relevant studies indexed exclusively in other databases, such as SPORTDiscus, PsycINFO, CINAHL, or EMBASE, may not have been identified. The review was limited to peer-reviewed RCTs and did not include grey literature; therefore, unpublished or non-peer-reviewed studies, including studies with null or negative findings, may have been missed, potentially contributing to publication bias. The restriction to English-language publications may have introduced language bias and potentially resulted in the omission of relevant evidence published in other languages. Furthermore, sex distribution was not explicitly reported in two included studies [76,77], limiting the complete characterisation of the sex composition of the included athlete populations. Publication bias could not be formally assessed because this scoping review did not undertake a quantitative meta-analysis or pool effect estimates. Risk of bias and certainty of evidence were also not formally assessed, consistent with the evidence-mapping purpose of this scoping review. In addition, the synthesis relied primarily on the direction and statistical significance of reported findings rather than standardised effect estimates, which limits interpretation of the magnitude and precision of intervention effects. Although data were independently extracted and discrepancies were resolved through reviewer discussion, an inter-rater reliability statistic was not calculated, and potential extraction inconsistencies cannot therefore be completely excluded. In addition, cost-effectiveness was not assessed, as economic outcomes were outside the predefined scope of this review and were not consistently reported in the included RCTs. Moreover, adverse events were not systematically reported across the included RCTs, limiting conclusions regarding the safety and tolerability of MBIs among athletes. The practical feasibility and real-world implementation of MBIs were not systematically assessed across the included RCTs.

The limitations create a clear agenda for future research. To produce reliable and replicable estimates of effects across diverse athletic populations (elite athletes, youth athletes, para-athletes, and athletes from low- and middle-income countries that are virtually under-represented in the current evidence base), adequately powered, multi-site RCTs with pre-registered protocols and a priori sample size calculations are urgently needed [94,95]. So, future trials should use multimodal outcome measures that integrate validated self-report measures with psychophysiological measures, such as cortisol, HRV, and neuroimaging measures, to enhance construct validity and minimise measurement bias [86,96,97]. Establishing a Core Outcome Set for athlete psychological well-being research may represent an important step toward improving the standardisation of outcome reporting, enabling meaningful cross-trial comparisons and prospective individual participant data meta-analyses [98].

## 5. Conclusions

This scoping review sought to map empirical evidence from RCTs examining the effects and patterns of MBIs on psychological well-being among athletes. The findings suggest that MBIs may provide beneficial effects across several psychological well-being outcomes among athletes; however, the magnitude and consistency of these effects vary across interventions, outcomes, and athlete populations. The evidence also suggests potential benefits in reducing psychological distress and improving cognitive aspects of anxiety, self-regulation, and attention. Some studies also reported beneficial effects when MBIs were compared with active control conditions, including relaxation and PST, although the comparative effects of MBIs remain uncertain. Given the relatively small sample sizes, heterogeneity in intervention protocols and outcome measures, and mixed findings across studies, these results should be interpreted cautiously. Further well-designed, adequately powered RCTs are needed to clarify the effects, optimal dose, comparative effects, and longer-term outcomes of MBIs in diverse athletic populations.

## Figures and Tables

**Table 1:** PCC framework.

Element	Definition
Population	Athletes who are trained, competitive, collegiate/varsity, or elite in any sport (team or individual); athletes aged 16 years or older, as defined by the study authors.
Concept	Mindfulness-based interventions (e.g., Mindfulness-Based Stress Reduction, Mindfulness-Based Cognitive Therapy, mindfulness training/practice; meditation modalities including body scan, breath awareness, focused attention, open monitoring, loving-kindness/compassion), delivered as standalone or sport-adapted programs. Outcomes oriented to psychological well-being.
Context	Sport and exercise settings (laboratory or field testing, training environments, collegiate/high-performance centres, competition phases); no geographic restriction.

**Table 2:** Inclusion criteria and Exclusion criteria.

Domain	Inclusion Criteria	Exclusion Criteria
Population	Trained, competitive, collegiate/varsity, or elite athletes in any sport (team or individual); athletes aged 16 years or older.	Non-athletes (general students, recreational-only, or sedentary populations), clinical samples unrelated to sport, and pediatric participants, unless classified as athletes.
Intervention/Concept	Mindfulness-based interventions (e.g., Mindfulness-Based Stress Reduction, Mindfulness-Based Cognitive Therapy, mindfulness training, meditation, body scan, breath awareness, focused attention, open monitoring, loving-kindness, compassion meditation); primary or clearly isolated component; any delivery format.	Non-mindfulness interventions (e.g., relaxation, Cognitive Behavioral Therapy without mindfulness, acceptance-based without explicit mindfulness); multi-component if the effect of mindfulness is not isolated; minor mindfulness elements only; mindfulness intervention not clearly mentioned.
Outcomes	At least one psychological well-being outcome (e.g., anxiety, depression, stress, mood, resilience, mental toughness, mindfulness, self-compassion, quality of life, sleep related to mental health).	No psychological well-being outcome (e.g., only physiological or performance outcomes, no mental health measurement), qualitative-only if not included.
Study Design	Randomised controlled trials (e.g., parallel, crossover, cluster), or author-classified randomised controlled clinical trials.	Non-randomised studies, quasi-experimental designs, observational or single-arm studies, protocols, qualitative-only, opinion/editorial/letter, animal/in vitro. Purely qualitative studies. Mixed-methods studies in which qualitative data collection and analysis are integral to the design (e.g., convergent parallel, explanatory sequential, or exploratory sequential mixed-methods).
Context	Sport/exercise settings, including laboratory/field testing, athlete training, or competition environments.	Non-athlete population context, general public, or clinical settings without an athlete context.

**Table 3:** Participant characteristics of the included studies.

Sl. No.	Author (Year)	Country	Objectives	Recruitment Location	Study Design	Sample Size	Groups (Intervention and Control)	Analysed Sample Size	Sex	Age (Mean Age)	Training Characteristics	Follow-Up	Sport/Population
1	Ajilchi et al. (2019) [69]	Iran & Australia	Effects of mindfulness training on mental toughness and emotional intelligence in basketball.	Tehran, Iran	Parallel-group pre-test and post-test RCT	*n* = 30	Intervention *n* = 15; Control *n* = 15	*n* = 30 (Intervention *n* = 15; control *n* = 15)	All male	23.5 years	Experience: 1.5–1.6 years; Frequency: ≥2 days/week	Not reported.	Amateur basketball players
2	Pakulanon & Petviset (2025) [70]	Thailand	Effects of imagery training and mindfulness meditation training on competitive anxiety and HRV in university athletes.	Chiangrai, Thailand	Three-arm RCT with block randomisation	*n* = 30	Imagery *n* = 10; mindfulness *n* = 10; Control *n* = 10	*n* = 30 (Imagery *n* = 10; mindfulness *n* = 10; control *n* = 10)	Males and females	21.3 years	Frequency: ≥2 days/week for 6 months; Competition: ≥1 competition in previous 12 months	Not reported.	University athletes in badminton, athletics, football, and swimming.
3	Hut et al. (2023) [71]	USA	Effects of MSPE compared with PST on collegiate student-athletes.	Not reported.	Parallel-group RCT	*n* = 32	MSPE *n* = 16; PST *n* = 16	*n* = 24 MSPE *n* = 15; PST *n* = 9)	Males and females	19.52 years	Not reported.	Not reported.	NCAA Division III collegiate track and field athletes
4	Ronkainen et al. (2026) [72]	Finland	Effects of a sport-specific ACT group intervention on mental well-being, stress, recovery, self-esteem, depression, and psychological flexibility in athletes.	Not reported.	Randomised controlled superiority trial with pre- and post-measurements	*n* = 95	ACT *n* = 49; WL Control *n* = 46	*n* = 93 (ACT *n* = 47; WL control *n* = 46)	Males and females	19.9 years	Volume: 15.11 h/week; Level: National	Not reported.	National-level athletes in athletics, basketball
5	Barbry et al. (2025) [73]	France	Effects of BMM combined with HIIT-based running on mindfulness skills and well-being in trained runners.	Not reported.	Parallel-group RCT with active control	*n* = 65	BMM *n* = 33; PST *n* = 32	*n* = 55 (BMM *n* = 30; PST *n* = 25)	Males and females	41.7 years	Level: Tier 2–3; Frequency: ~3 sessions/week	Not reported.	Competitive runners in athletics
6	Röthlin et al. (2020) [74]	Switzerland	Differential and shared effects of PST and mindfulness training on psychological variables related to athletic performance.	Magglingen, Switzerland	Three-group RCT	*n* = 95	MT *n* = 32; PST *n* = 32; WL *n* = 31	*n* = 95 (MT *n* = 32; PST *n* = 32; WL *n* = 31)	Males and females	24.43 years	Volume: ≥4 h/week eligibility; mean 7.94 h/week	Not reported.	Competitive athletes in tennis, curling, floorball, badminton
7	Ajilchi et al. (2022) [75]	Iran & Australia	Effects of MAC training on mindfulness, mental toughness, and psychological well-being in female athletes.	Tehran, Iran	Parallel-group, pre–post randomised controlled pilot trial with 2-month follow-up	*n* = 45	Intervention *n* = 23; Control *n* = 22	*n* = 42 (Intervention *n* = 21; control *n* = 21)	all female	21.55 years	Frequency: 3.78 days/week; Level: National	8 weeks	Female university athletes in swimming, badminton, and table tennis
8	Qian et al. (2026) [76]	China	Effects of a 16-week mindfulness intervention on cognitive performance, cerebral oxygenation, emotional state, salivary cortisol, and mental fatigue in table tennis athletes.	Beijing, China	Single-blind randomised crossover design	*n* = 90	mindfulness *n* = 45; Control *n* = 45	*n* = 71 (mindfulness *n* = 39; control *n* = 32)	Not explicitly stated	19–26 years	Not reported.	Not reported.	College table tennis athletes
9	Sabzevari et al. (2023) [77]	Iran & Brazil	Effects of a MAC-based approach on rumination, cognitive flexibility, and sports performance in elite beach soccer players.	Not reported	Parallel-group RCT with follow-up	*n* = 40	Intervention *n* = 20; Control *n* = 20	*n* = 34 (Intervention *n* = 19; control *n* = 15)	Not explicitly stated	25.3 years	Experience: ≥6 years; Level: National team/Premier League of beach soccer.	8 weeks	Elite beach soccer players from the Premier League of beach soccer
10	Scott-Hamilton et al. (2016) [78]	Australia	Effects of an 8-week MBI on mindfulness, flow, sport anxiety, and pessimistic attributions in competitive cyclists.	New England, Australia	RCT with wait-list control	*n* = 60	Intervention *n* = 30; Control *n* = 30	*n* = 47 (Intervention *n* = 27; control *n* = 20)	Males and females	39.8 years	Volume: 1–10 h/week; Competition: Most ≥1/week	Not reported.	Competitive road and mountain bike cyclists
11	Yu et al. (2024) [79]	China	Effects of a 7-week MAIC mindfulness training program on competition state anxiety and mindfulness in Chinese sprinters.	Jilin, China	Single-blind RCT with 2 × 3 mixed design	*n* = 24	mindfulness *n* = 12; Control *n* = 12	*n* = 24 (mindfulness *n* = 12; control *n* = 12)	Males and females	22.46 years	Experience: 11 < 6 years; 13 ≥ 6 years	Not reported.	Competitive sprinters are eligible for the 100 m dash at the 21st National Collegiate Athletics Championships.
12	Josefsson et al. (2019) [80]	Sweden	Effects of MAC intervention on emotion regulation, sport-specific mindfulness, and self-rated athletic training performance compared with PST.	Southwest area of Sweden	Parallel-group RCT	*n* = 69	MAC *n* = 36; PST control *n* = 33	*n* = 68 (MAC *n* = 36; PST control *n* = 32)	Males and females	20.9 years	Competitive level: Local (n = 1), regional (n = 5), national (n = 32), international (n = 26)	Not reported.	Competitive elite athletes in floorball, golf, football (soccer), cycling, wrestling
13	Mohebi et al. (2021) [81]	Iran, USA & Switzerland	Effects of a MAC program on self-compassion, grit, and mindfulness in elite female athletes.	Tehran, Iran	Parallel RCT	*n* = 40	MAC *n* = 20; Control *n* = 20	*n* = 40 (MAC *n* = 20; Control *n* = 20)	All female	22.22 years	Experience: 8.30 years; Frequency: ≥3 sessions/week; Level: National	4 weeks	Elite female athletes at the national competition level, from various team and individual sports
14	Glass et al. (2019) [82]	USA	Effects of a 6-session MSPE program on well-being, sport-related psychological factors, and performance in collegiate athletes.	Not reported	Parallel RCT with wait-list control	*n* = 57	MSPE *n* = 28; WL control = 29	*n* = 52 (MSPE *n* = 23; WL control = 29)	Males and females	19.32 years	Not reported.	20–24 weeks	NCAA Division III collegiate student-athlete in cross-country/track, swimming, tennis, lacrosse, field hockey, soccer, baseball, football, and volleyball.
15	Gao et al. (2024) [83]	China & USA	Effects of a therapist-guided, smartphone-delivered mindfulness intervention on anxiety and mindfulness in college athletes.	Shanghai, China	Parallel-group RCT	*n* = 288	Intervention *n* = 150; Control *n* = 138	*n* = 288 (Intervention *n* = 150; Control *n* = 138)	Males and females	19.4 years	Not reported.	Not reported.	College student athletes in soccer, volleyball, cheerleading, basketball, badminton, bridge, chess, fencing, golf, table tennis, skipping rope, swimming, taekwondo, tennis
16	Staiano et al. (2026) [84]	Spain, Denmark & Ecuador	Effects of a 6-week MBI on cognitive and sport-specific physical performance in elite handball players.	Not reported	Randomised controlled pre-test–post-test training study	*n* = 91	MBI *n* = 46; Control *n* = 45	*n* = 79 (MBI *n* = 40; Control *n* = 39	Males and females	20.1 years	Level: Highly trained/elite; Frequency: 5 supervised sessions/week; Duration: 6 weeks	Not reported.	Handball players are classified as highly trained/elite.
17	Macdougall et al. (2019) [85]	Australia	Effects of an 8-session MAC program integrated with motivational interviewing on global well-being in Para athletes.	Victoria, Australia	Pilot RCT with concealed allocation	*n* = 18	Intervention *n* = 9; Control *n* = 9	*n* = 18 (Intervention *n* = 9; Control *n* = 9)	Males and females	32.5 years	Experience: <2, 2–5, 5–10, and >10 years; each 22–33%, except >10 years (11%)	Not reported.	Elite athletes in Paralympic sports

Note: RCT = Randomised controlled trial; MSPE = Mindful sport performance enhancement; MAC = Mindfulness-acceptance-commitment; MBI = Mindfulness-based intervention; MAIC = Mindfulness Acceptance Insight Commitment; PST = Psychological skills training; BMM = Brief mindfulness meditation; HIIT = High-intensity interval training; NCAA = National Collegiate Athletic Association; MT = mindfulness training; WL = wait-list; HRV = Heart rate variability.

**Table 4:** Intervention characteristics of the included studies.

Sl. No.	Author (Year)	Intervention Characteristics	Comparator	Outcomes Measured	Key Findings
1	Ajilchi et al. (2019) [69]	MSPE; 6 weeks; weekly 90 min group sessions with home practice and emailed mindfulness exercises	Passive control (usual coaching only)	Outcomes: Mindfulness; mental toughness; emotional intelligence. Instruments: MSPQ; Mental Toughness Questionnaire; Self-rated Emotional Intelligence Scale.	Significant improvements in mindfulness, mental toughness, and emotional intelligence compared with controls
2	Pakulanon & Petviset (2025) [70]	Mindfulness meditation training; 8 weeks; 3 sessions/week; 30 min/session	Passive control (stress reduction brochure); active comparator: imagery training	Outcomes: Competitive state anxiety (somatic anxiety, cognitive anxiety, self-confidence). Instrument: CSAI-2R.	Significant reduction in cognitive anxiety; no significant changes in somatic anxiety, self-confidence, or HRV
3	Hut et al. (2023) [71]	MSPE; 6 weeks; 1 h/week; in-person group sessions using train-the-trainer model	Active control receiving PST	Outcomes: Sport anxiety; flow; depression; anxiety; stress; mindfulness; emotion regulation; experiential avoidance; sport satisfaction; enjoyment; perceived influence of negative internal/external states on performance. Instruments: SDFS-2; CDFS-2; DASS-21; SAS-2; PHLMS; MIS; DERS-SF; BEAQ; SRF; PEQ.	Both groups improved sport anxiety and enjoyment; MSPE showed greater improvement in satisfaction with sport performance.
4	Ronkainen et al. (2026) [72]	Sport-specific ACT; 6 weeks; weekly 60 min sessions (first session 75 min); face-to-face group sessions with home exercises	Wait-list passive control	Outcomes: Mental well-being; stress; experienced recovery; self-esteem; depression; psychological flexibility. Instruments: MHC-SF; PSS; REQ; RSES; PHQ-9; PFSS; VAMS; AMQ; ELS.	Improved mental well-being and reduced stress; increased psychological flexibility; no significant effects on depression, self-esteem, or recovery
5	Barbry et al. (2025) [73]	BMM—“FeelTheRun”; 8 weeks; 3 sessions/week; 5–12 min/session plus home practice	Active control receiving running information and one PMR session	Outcomes: Mindfulness; well-being manifestations. Instruments: MIS; Diagnofeel questionnaire.	Improved refocusing ability, social relations, and pleasure regulation; no significant effects on acceptance
6	Röthlin et al. (2020) [74]	Mindfulness Training; PST: 4 weeks; four 90 min group workshops; home assignments and 10–12 min daily audio-guided exercises.	Wait-list control and active PST comparator	Outcomes: Handling of emotions; experiential avoidance; emotional acceptance; attention control; cognitive interference; decentering; dealing with failure (action vs. state orientation); use of psychological skills. Instruments: Mindfulness; Use of Mental Strategies; Acceptance of Emotions; Experiential Avoidance; Attention Control in Training and Competition; Cognitive Interference; Decentering; Action Orientation vs. State Orientation after Failure; Ability to Prevent Emotions from Interfering with Performance.	Both Mindfulness Training and PST improved emotion regulation and attention; Mindfulness Training uniquely reduced experiential avoidance.
7	Ajilchi et al. (2022) [75]	MAC programme; 7 weeks; weekly 45 min sessions with home practice ≥3 days/week	Passive control (usual sport coaching)	Outcomes: Mindfulness; mental toughness; psychological well-being. Instruments: MIS; SMTQ; PWB Scale.	Significant improvements in mindfulness, mental toughness, and psychological well-being Improvements in mental toughness and psychological well-being were maintained at the 2-month follow-up.
8	Qian et al. (2026) [76]	Mindfulness intervention; 16 weeks; 8 min/session; 4 sessions/week after training	Passive control	Outcomes: Mindfulness; emotional state; mental fatigue. Instruments: FFMQ; cognitive tasks; STAI; BDI; SHS; Borg’s RPE.	Improved cognition, emotional state, reduced fatigue, and lower cortisol
9	Sabzevari et al. (2023) [77]	MAC-based intervention; 7 weeks; 60–90 min per session, weekly sessions plus daily homework	Passive control	Outcomes: Rumination; cognitive flexibility; sport performance. Instruments: RRS; CFI; Charbonneau Sports Performance Questionnaire.	Significant improvements in cognitive flexibility, sport performance, and reduced rumination and maintained at 2-month follow-up.
10	Scott-Hamilton et al. (2016) [78]	Sport-specific mindfulness training based on MiCBT with mindful spin-bike component; 8 weeks; workshops, daily meditation, weekly mindful spin-bike sessions	Passive control	Outcomes: Mindfulness; flow in sport; flow composites; sport anxiety; sport pessimistic attributions. Instruments: FFMQ; DFS-2; SAS-2; SASS.	Improved mindfulness, flow, and reduced pessimistic attributions; mixed findings for anxiety
11	Yu et al. (2024) [79]	MAIC training; 7 weeks; weekly 60 min sessions; 15 min daily home breathing exercises after training	Active control receiving psychological guidance	Outcomes: Competition state anxiety; mindfulness. Instruments: FFMQ; Competition State Anxiety Scale.	Significant improvements in mindfulness and reductions in competition state anxiety
12	Josefsson et al. (2019) [80]	MAC protocol; 7 weeks; weekly 50 min in-person group sessions	Active control receiving PST	Outcomes: Dispositional athletic mindfulness; emotional regulation; self-rated training performance; adherence. Instruments: AMQ; DERS; single-item performance scale; self-reported attachment.	Greater improvements in mindfulness, emotion regulation, and perceived performance compared with PST
13	Mohebi et al. (2021) [81]	MAC training; 7 consecutive weeks; weekly 60 min sessions delivered by trained sport psychologists	Active control receiving sport psychology education	Outcomes: Mindfulness; self-compassion; grit. Instruments: MIS; SCS-SF; SG-S.	Significant improvements in mindfulness, self-compassion, and grit; effects maintained at follow-up
14	Glass et al. (2019) [82]	MSPE; 6 weeks; weekly 75 min group sessions	Passive wait-list control	Outcomes: Depression; anxiety; stress; life satisfaction; trait mindfulness; psychological inflexibility; flow in sport; sport anxiety; self-rated sport performance; coach-rated sport performance. Instruments: DASS-21; SWLS; FFMQ; AAQ-II; DFS-2; SAS; SRF; CRF.	Improvements in flow, mindfulness, life satisfaction, and sport performance; reduced worry; maintained at 6-month follow-up
15	Gao et al. (2024) [83]	Smartphone-delivered MBSR; 43 days (~6.1 weeks); daily 15 min guided meditation via WeChat with therapist interaction	Active control receiving mental health education materials	Outcomes: Trait anxiety; competition anxiety; mindfulness. Instruments: Athlete anxiety scales; FFMQ.	No significant effects on anxiety outcomes; near-significant improvement in mindfulness observation facet
16	Staiano et al. (2026) [84]	Mindfulness intervention using mPEAK + Headspace; 6 weeks; 2.5 h/week plus 20 min/day guided mindfulness	Active control	Outcomes: Mind-wandering; response inhibition; mental fatigue; cognitive load; subjective demand; dispositional and multifactorial mindfulness. Instruments: SART; Stroop; MVAS; NASA-TLX; MAAS; FFMAQ.	Improved cognitive performance, dispositional mindfulness, and reduced mental demand, fatigue,
17	Macdougall et al. (2019) [85]	MAC programme within motivational interviewing framework; 8 weeks; weekly 60 min individual face-to-face sessions	Passive control (usual activities)	Outcomes: Subjective, psychological, social, and physical well-being; mindfulness; experiential acceptance; psychological flexibility; performance. Instruments: SPANE; SWLS; Ryff’s PWB Scale; Keyes SWBQ; Athlete Burnout Measure; Pain Catastrophising Scale; CAMS-R; AAQ-II.	Significant improvements in life satisfaction, personal growth, social relations, and reduced pain perceptions.

Note: ACT = Acceptance and Commitment Therapy; MAC = Mindfulness-Acceptance-Commitment; MAIC = Mindfulness Acceptance Insight Commitment; MSPE = Mindful sport performance enhancement; MBSR = Mindfulness-Based Stress Reduction; BMM = Brief Mindfulness Meditation; PST = Psychological skills training; HRV = Heart rate variability; MiCBT = Mindfulness-integrated Cognitive Behaviour Therapy.

## Data Availability

No new data were created or analysed in this study. Data sharing is not applicable to this article.

## Supplementary Materials

The following supporting information can be downloaded at: https://www.mdpi.com/article/10.3390/healthcare14182899/s1, Supplementary S1: Preferred Reporting Items for Systematic Reviews and Meta-Analyses extension for Scoping Reviews (PRISMA-ScR) Checklist; Supplementary S2: Search Strategy.
